# Pharmacology-based ranking of anti-cancer drugs to guide clinical development of cancer immunotherapy combinations

**DOI:** 10.1186/s13046-021-02111-5

**Published:** 2021-10-01

**Authors:** Vincent Lemaire, Colby S. Shemesh, Anand Rotte

**Affiliations:** 1 Department of Clinical Pharmacology, Genentech Inc, 1 DNA Way, South San Francisco, CA 94080 USA; 2 Independent Consultant, Santa Clara, USA; 3 Current address: Clinical and Regulatory Affairs, Arcellx, Gaithersburg, USA

**Keywords:** Clinical trials, Cancer immunotherapy, Combination development, Cancer, Pharmacology

## Abstract

The success of antibodies targeting Programmed cell death protein 1 (PD-1) and its ligand L1 (PD-L1) in cancer treatment and the need for improving response rates has led to an increased demand for the development of combination therapies with anti-PD-1/PD-L1 blockers as a backbone. As more and more drugs with translational potential are identified, the number of clinical trials evaluating combinations has increased considerably and the demand to prioritize combinations having potential for success over the ones that are unlikely to be successful is rising. This review aims to address the unmet need to prioritize cancer immunotherapy combinations through comprehensive search of potential drugs and ranking them based on their mechanism of action, clinical efficacy and safety. As lung cancer is one of the most frequently studied cancer types, combinations that showed potential for the treatment of lung cancer were prioritized. A literature search was performed to identify drugs with potential in combination with PD-1/PD-L1 blockers and the drugs were ranked based on their mechanism of action and known clinical efficacy. Nineteen drugs or drug classes were identified from an internal list of lead molecules and were scored for their clinical potential. Efficacy and safety data from pivotal studies was summarized for the selected drugs. Further, overlap of mechanisms of action and adverse events was visualized using a heat map illustration to help screen drugs for combinations. The quantitative scoring methodology provided in this review could serve as a template for preliminary ranking of novel combinations.

**Supplementary Information:**

The online version contains supplementary material available at 10.1186/s13046-021-02111-5.

## Background

In 2020, cancer remained the second leading cause of death with 2 million new cancer diagnoses and over 0.5 million cancer deaths projected in the United States alone [1]. As a new standard of care validated in at least 17 different types of cancer including 2 tissue-agnostic indications, anti-programmed cell death protein 1 (PD-1) and its ligand L1 (PD-L1)-targeted checkpoint inhibitors have harnessed the immune system to radically combat many cancers. Based on durable responses seen across several tumor types, these agents have become a backbone to the largest clinical trial programs in history, raising the bar for clinical efficacy of new therapeutics [2]. Despite growing interest and high eligibility for patients to receive these agents, the percentage of patients expected to respond remains modest for the most part, ranging from 12 to 65 % [3, 4]. This has motivated investigators to develop drugs that go beyond checkpoint blockade and additional standard of care therapies to target other pathways in combination approaches that overcome primary resistance, deepen existing responses, and rescue patients progressing due to secondary resistance. While the research in combination therapy resulted in approval of PD-1/PD-L1 targeting drug combinations with cytotoxic T-lymphocyte antigen 4 (CTLA-4) blockers, chemotherapy, drugs targeting vascular endothelial growth factor (VEGF) and drugs targeting intracellular kinases for over half-a-dozen types of cancer (Supplementary Table S1), additional studies are needed to further improve the response rates and to treat other types of cancer.

The current search for life-altering combination therapies benefiting a broader number of patients across indications is immense and skyrocketing. As of September 2020, there were 2,949 clinical trials of anti PD-1/PD-L1 agents in combination with hundreds of targets to address multiple mechanisms of immune escape. The existing combination trials are estimated to enroll more than half a million patients [2]. Many of these clinical trials evaluate combinations empirically without sufficient knowledge, or with inconsistent understanding regarding translatability of activity and toxicity amongst a plethora of diverse and novel targets. Many of the approaches are of limited utility and are doomed to fail, contributing to the low success rates of anticancer drug development where only 7 % of drugs tested in phase I are expected to reach licensure [5, 6]. Moreover, despite the potential for a greater immune response with combination strategies, many of these run the risk for harmful adverse events and increased, unexpected, overlapping, and synergistic toxicity that could be serious and even lethal [7]. While safety is prioritized, immense resources and careful consideration are required for the evaluation of organ function, laboratory abnormalities, and other complications [8–12]. For these reasons, there is a need to refine approaches that offer less risk, with objective strategic prioritization of resources and streamlining of efforts to support higher success rates in signal seeking combination trials. Failure to de-prioritize combinations that have questionable benefit at an increased risk is a burdensome disservice to patients that comes at a high cost to the society [5, 13–16]. Ranking of combinations should be multifactorial, including in-depth assessments for the molecular and immune pathways of drugs that can produce desired effects on immune cells [17, 18], and better understanding of relative benefit/risk characteristics.

### Challenges in development of combination therapies

As listed in Table 1, the complexity of combination development of cancer immunotherapies is daunting, and currently benefit of long-term disease control appears possible in roughly only 20 % of patients with checkpoint inhibitors [19]. Further, a variety of responses are seen in patients with the same therapy, along with dissimilar responses by tumor type given high disease heterogeneity and varying tumor immune phenotype [20]. For an immunotherapy to be effective, effector immune cells must traffic to the tumor, infiltrate stroma, and overcome a hostile immunosuppressive tumor microenvironment [21]. Preclinical models poorly predict the success of clinical candidates and translating basic research and preclinical findings to optimal clinical combinations remains arduous [20]. Mechanisms of primary and acquired resistance during treatment with immunotherapies remain to be elucidated. Patient selection and stratification based on relevant biomarkers has been substantially limited and near non-existent; a study of planned clinical trials in 2019 showed that less than 10 % of the studies required biomarkers for enrollment [22]. A very limited number of biomarkers are linked to higher chance of response to anti-PD(L)1-based immunotherapies at the population level, e.g., PD-L1 and tumor mutational burden (TMB) [23]. And, to date, even the most sophisticated biomarker relationships are not entirely predictive of response in individual patients. Benefits for the average patient may not help an individual patient and, although, it is clear that immunotherapies have revolutionized oncology therapy, they have fallen short of widespread successes [24]. For more than 75 % of patients with cancer, the opportunity to participate in a clinical trial is non-existent, due to a lack of local trials for or due to trial ineligibility [25]. For testing of new immunotherapy combinations, there is a fierce competition to recruit any remaining eligible patients, demonstrated by dwindling enrollment rates in the United States [26]. Thus there is an urgent need for clearer mechanistic rationale for prioritizing new clinical trials of combination cancer immunotherapies [24], to allow for better understanding the complex interaction of drugs on a patient’s immune system, the interplay of immune cells and cancer cells, and for prediction of adverse effects.

**Table 1:** Examples of key issues impacting development of cancer immunotherapy combinations

Key Issues
1. Exponential increase in number of potential targets/molecules for development
2. Complex mechanisms of action, which may or may not have synergistic or additive interaction
3. Need for guidance on dose, regimen and sequence of the combination
4. Possibility of higher incidence of serious adverse events
5. Possibility of combination being effective only in hematological tumors or in solid tumors
6. Time lags in getting early data on dose, efficacy and safety
7. Lack of clinical data to propose rational and quantitative assessments
8. Need for strategies to apply combinations with the goal of turning ‘cold’ tumors into ‘hot’
9. Competitive pressure and speed of development

### Review objectives

The aim of this review is to support the development of immunotherapy combinations and address the challenge of identification of potential drugs for combinations. Since the efficacy of immunotherapy as monotherapy and combinations can vary with cancer type, we focused our review on lung cancer, which is one of the most frequently studied cancer types. We propose a ranking system based on the pharmacology of drugs and apply the method to rank the promising drugs for immunotherapy combinations for lung cancer treatment. We present an objective, and comprehensive review of select targets that may serve as a rational basis and illustrative tool to help narrow the selection. We mined and incorporated a wide range of drugs and focused on several of the most interesting targets of high potential for combination immunotherapy. This review is organized as follows: first, the methods section describes the selection of the initial list of drugs and ranking methodology used to select the lead molecules; next, stages involved in selecting the final set of drugs are described; then, the risk-benefit profile with emphasis on overlap of mechanisms of action and safety profile are described; and lastly, the strengths and limitations of the review are discussed along with the summary of the review.

## Methods

### Selection of initial list of drugs/molecular targets

**Fig. 1:**
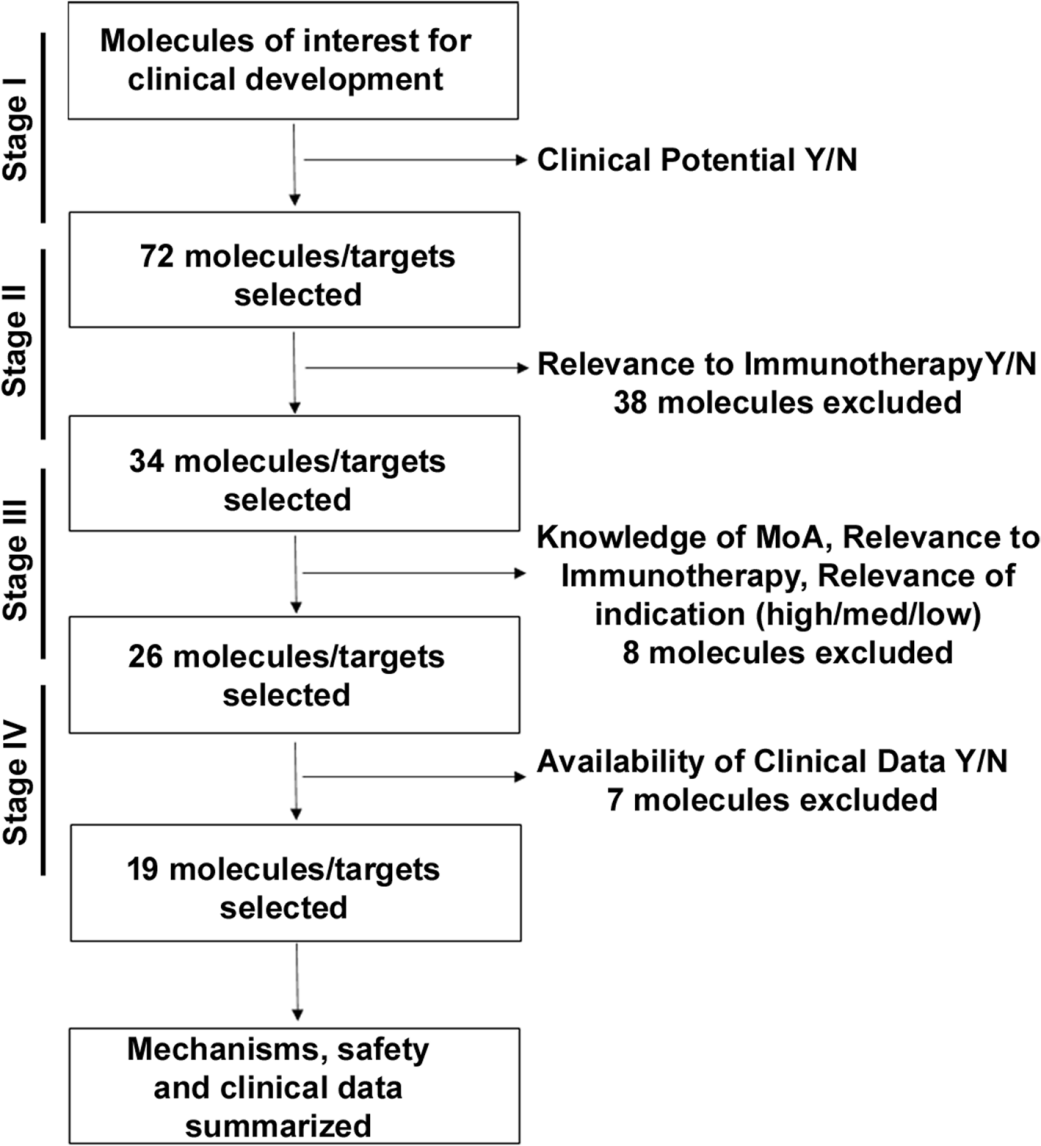
Flow chart of the search and elimination process for selection of drugs of interest. *PD-1/PD-L1 class of drugs, were preselected as they are considered as the backbone for combination studies. The final number of drugs selected is 20 (19 + anti-PD-1/PD-L1)

Our approach is illustrated by the flow chart displayed in Fig. 1. A list of drugs and molecular targets was provided from the internal clinical development programs as a starting point. The main criteria for selecting the drugs during our screening were, in order of importance: (1) Drugs for which there is enough information on their mode of action, especially relevant to their action on the immune system; (2) Drugs that have complementary mechanisms of action in cancer immunity and that could potentially work well in combination; (3) Drugs for which there is enough data to derive a preliminary characterization of their efficacy and safety/toxicity profile in human; and lastly, (4) Drugs that have already been approved as monotherapy or combination therapy and with significant information on safety. Drugs already tested in combinations, or applicable to a larger number of cancer types (only solid tumors), carry higher value in our selection process. Points 3 and 4 imply that we limit our analysis to drugs that have already been tested in the clinic. There are several reasons for doing that. First, as pointed out above, identifying combinations that may succeed in the clinic is extremely challenging. Doing so with no monotherapy clinical efficacy and/or safety data would make this analysis too speculative. Second, the vast majority of all combinations being evaluated in clinical trials at this time have previous clinical exposure as monotherapy agents. This is for mitigation of the risk of failure. Our approach proposes a more thorough and analytical assessment of drugs with existing clinical exposure before they are evaluated in combination in the clinic.

### Ranking methodology

Our initial screening was done for 72 drugs that were either already approved for clinical use or were in clinical development (Fig. 1). All 72 drugs were carefully assessed, scored, and ranked, based on the scoring criteria summarized in Table 2. While all the selected drugs had promising potential, the cross talk of their mechanism of action with immune response, granular knowledge of downstream pathways effected by the treatment and their efficacy in solid versus heme cancers varied widely. Therefore, drugs were mainly scored for their clinical potential, relevance to immunotherapy, relevance to indication, knowledge of the MOA, effect on immune response, availability of clinical efficacy data.

**Table 2:** Scoring methodology for the available information

	Yes/High (3)	Medium (2)	No/Low (1)
Stage I screening			
Clinical potential	Actively considered for clinical development with at least solid preclinical data	NA	Mainly in vitro data and < 5 studies showing preclinical evidence
Stage II screening			
Relevance to immunotherapy	Directly activate immune system or known to have significant indirect effects on immune system	NA	Not known to directly or indirectly activate immune system
Stage III screening			
Relevance of Indication	> 3 indications in solid tumors including NSCLC in clinical development	At least 3 indications in clinical development	< 3 indications in clinical development or heme indications only
Knowledge of MoA	Biology is clearly established with details of interactions at cellular and molecular levels	Biology is not clearly established with only few details on molecular interactions	Biology not known
Effects on immune response	Directly activate effector immune cells such as CD8 T-cells or NK cells	Act by stimulating the proliferation of immune cells or increasing the infiltration of immune cells into tumors	Indirectly activate immune system through antigen release
Stage IV screening			
Availability of data	Efficacy and safety data validated in multiple clinical studies	NA	Limited clinical or only preclinical efficacy and safety data
Scoring of clinical data			
Clinical efficacy data	Data available from combination with PD-1/PD-L1 blockers in solid tumors; Data from monotherapy in solid tumors and combination with chemotherapy in solid tumors	Only data from monotherapy; or only combination therapy in solid tumors is available	Only data from heme tumors and/or preclinical data is available
Other information summarized (not scored)			
Setting	Adjuvant +/- or Adv/Metastatic 1-2 L or Adv/Metastatic 3-4 L+		
Combination studies	Combination studies with CIT or targeted therapy or with standard of care (e.g. chemotherapy)		
Efficacy	Molecule/target active by itself in multiple indications or single indication or only in combination		
Safety	Whether AEs manageable and reversible with treatment cessation or require additional treatment for reversal or require hospitalization and aggressive treatment for reversal Grade 3 or more incidence		
Phase	Phase 1/2/3/4 or pre-clinical		
Time to read out	< 1 years or 1–3 years or > 3 years		

Abbreviation: *NA* not applicable

**Fig. 2:**
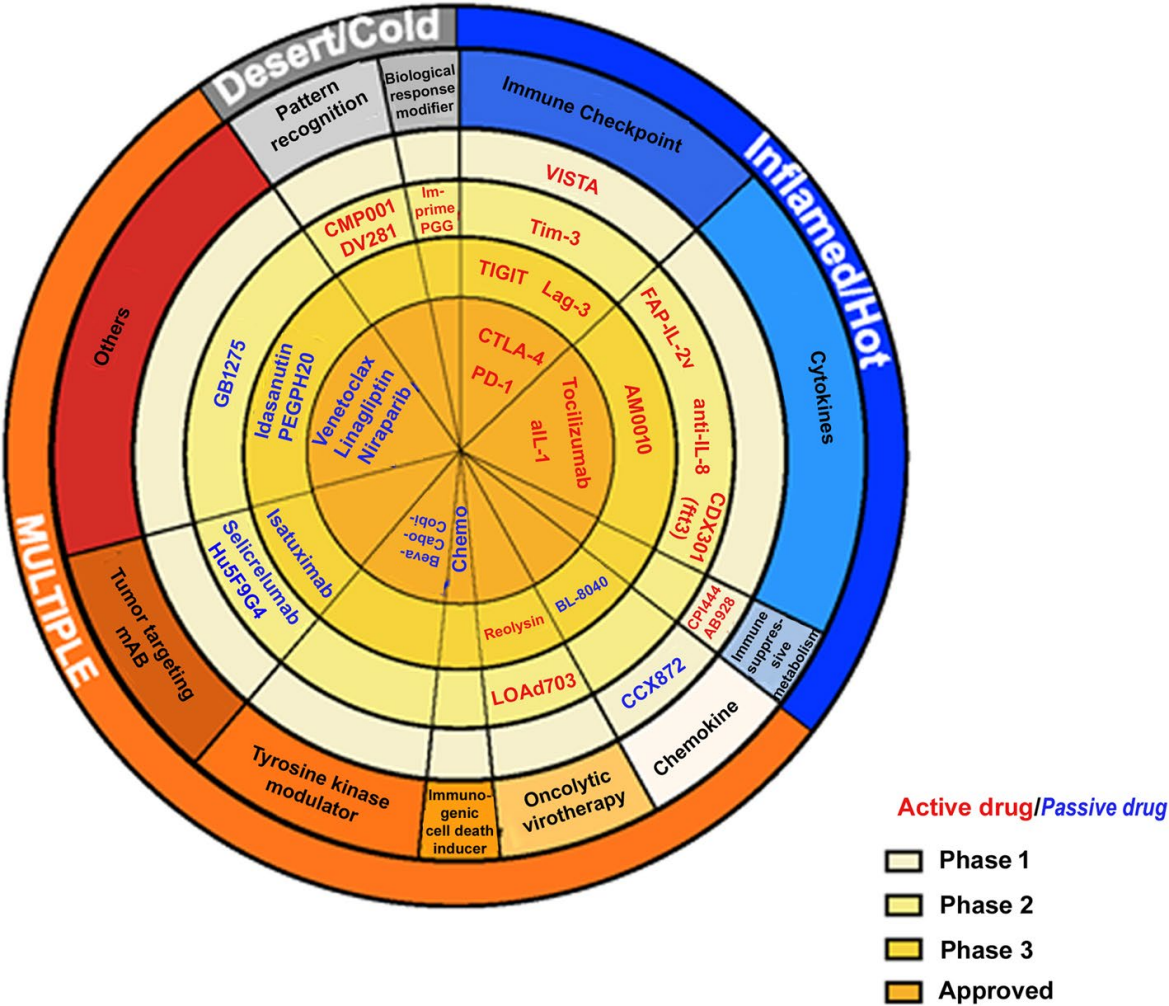
Pie chart of molecule classification. Coloring is used to identify tumor type, and mechanism of action, with the inner sectors representing development stage. Drugs are classified using a hybrid of multiple components including development stage, tumor type, mechanism of action, and are bucketed as passive or active immunotherapies based on immune response activation. Passive immunotherapies include molecules expressed in low levels; they rectify deficient immune system typically used for patients with impotent immune systems. These could include the monoclonal antibodies targeting malignant cells, adoptive transfer of immune cells, adjuvants, recombinant cytokines, inhibitors of signaling pathways, delivery of cytotoxins, activators of ADCC, tumor antigen targeting, and oncolytic viruses; which typically require multiple administrations to be efficient. Active immunotherapies are designed to activate effector function of immune cells. These include activation of endogenous and long-lasting immune responses including vaccines, blockade by checkpoint inhibitors, oncolytic viruses, immunomodulatory mAbs, immunostimulatory cytokine adjuvants to augment immunotherapy response, mAbs to proinflammatory cytokines, immunogenic cell death inducers such as chemotherapies, and pattern recognition receptor agonists.

Scores were derived from the compilation of detailed literature review and ranged from 1 (low) to 3 (high) in each category. After initial screening of drugs based on clinical anti-cancer potential (yes/no) in stage I (Table 2; Fig. 1), 72 drugs/class of drugs were selected. In stage II, all 72 drugs were scored based on relevance to immunotherapy (Table 2; Fig. 1) resulting in the elimination of 38 drugs. The remaining drugs of interest are displayed in Fig. 2, where the diversity in modes of action can easily be perceived. The drugs are arranged radially by their stage of clinical development (from phase 1 to approved). The categories of drugs/mode of action are further grouped by type of cancer-immune phenotypes they are most likely to be applicable to (inflamed/hot, desert/cold or multiple). Lastly, the drugs are labeled as ‘Active’ or ‘Passive’ depending on the way they engage the immune system, either directly (such as immune checkpoint inhibitors) or passively (such as chemotherapy).

The 34 remaining drugs were then scored in the Stage III screening (Supplementary Table S2) where 8 drugs were excluded (Fig. 1). Finally, in the stage IV screening, 7 more drugs were excluded and 19 drugs were selected. This list is displayed in the Supplementary Table S3 and represents the final step of the methodology flow chart provided in Fig. 1. A key point to be noted in our ranking model is that the availability of data for clinical activity or efficacy scoring is applied at the final stage, which prevents low scoring and eventual screening out of drugs that only have early efficacy data. Similarly, the number of indications in clinical development is only one component of the stage III scoring, allowing drugs in early stages of clinical development to be selected for evaluation of mechanism and safety overlap discussed in later sections. Finally, the current model gives more weight to drugs with clinical data in lung cancer and solid tumors. It can be adapted to other indications such as hematological cancers by weighting the data accordingly.

### Literature mining

Literature was collected by screening publicly available information using search portals such as PubMed, Google Scholar, Web of Science, as well as relevant conference websites including ASCO, ESMO, SITC, in addition to clinicaltrials.gov. In reviewing the literature on the mode of action of the drugs in our list, we focused our attention in identifying the most upstream effects of the drugs on the cancer/immune biology, as opposed to downstream effects. This approach allowed for an easier assessment of drugs that may lead to functional interactions in combination.

For our data collection effort of each of the drugs in Supplementary Table S3, we looked for 3 kinds of data, including: (1) Information on ongoing clinical trials that included the developmental phase, combination with other agents, indication, dose (if already established), and primary endpoints; (2) top-down data including baseline characteristics of patient population and clinical readouts from already published clinical trials; and (3) bottom-up data including data pertaining to the mechanism of action of the drugs. For top-down data, priority was given to data from phase 3 clinical trials, then phase 2 and phase 1. Response clinical endpoints such as the objective response rate (ORR) and the disease control rate (DCR) were recorded, as well as time to event endpoints including progression free survival (PFS) and overall survival (OS). All solid tumors were prioritized, and hematological tumors were not considered. For the bottom-up data, priority was given to collecting information on the effect of the drugs on immune cells in human, in plasma and in the tumor microenvironment (when available).

### Selected drugs

Out of the 20 drugs (including anti-PD-1/L1 antibodies) selected for our final summary and listed in Supplementary Table S3, 7 drugs/class of drugs are approved for the treatment of cancer (single/multiple types of cancer; monotherapy or combination with other anti-cancer therapies). All of them are currently under investigation or actively considered for combination with PD-1/PD-L1 blockers. Details of the molecular targets for each drug/drug class are included in Supplementary Table S2. In the following section, efficacy of combinations and potential for synergism are explained using the overlap of mechanism of action (MOA), while risks associated with combinations are explained using safety and serious/dose limiting adverse event (AE) overlap.

### Benefit risk profile

#### Efficacy: mechanism of action overlap and potential for synergistic effects

**Fig. 3:**
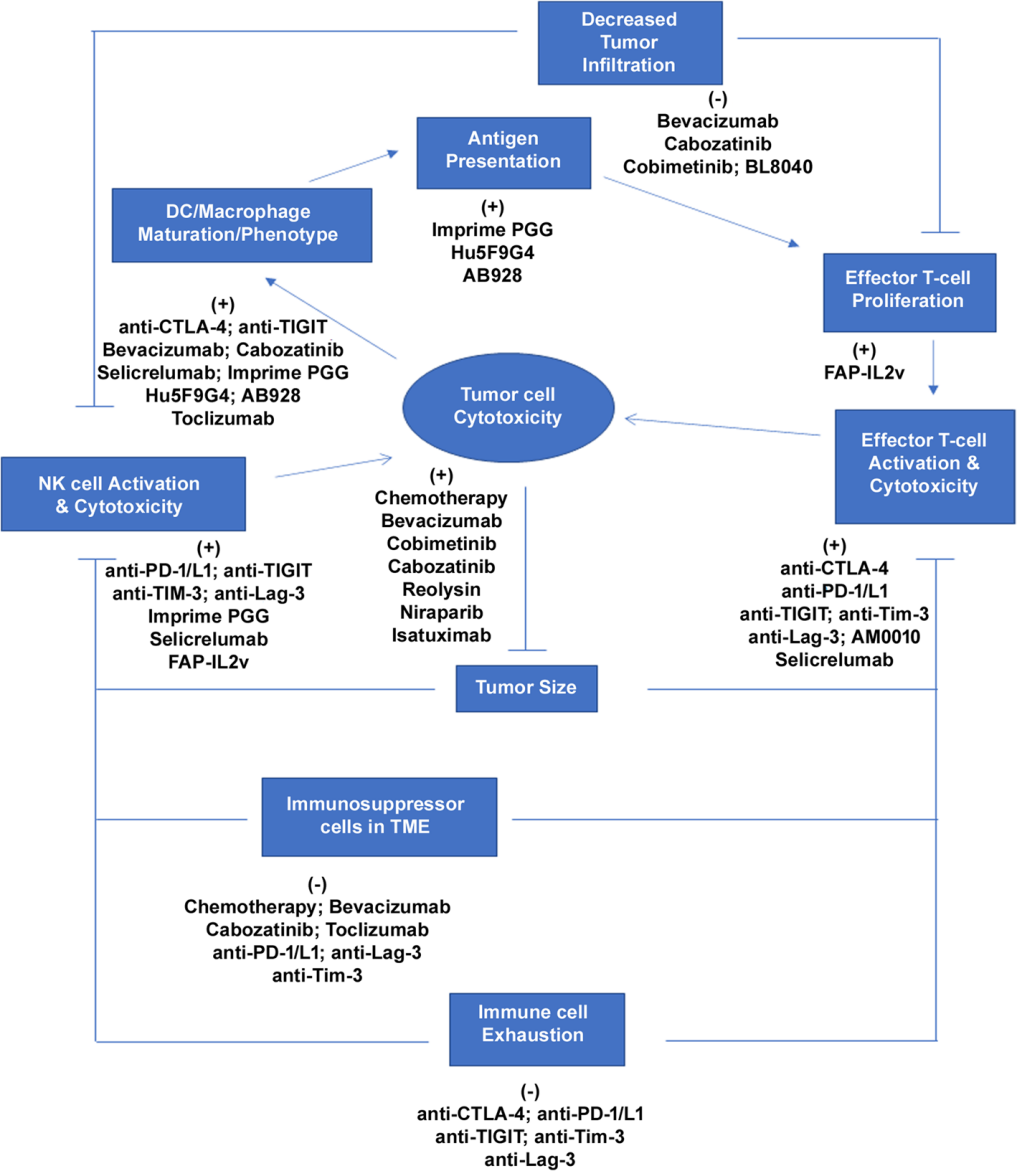
Flow chart showing the point of action for screened drugs. Tumor cell cytotoxicity is mainly achieved by effector T-cells and NK cells, which results in antigen release and reduction in tumor size. Release of antigens along with cellular components such as danger associated molecular patterns (DAMPs) result in maturation of DCs and macrophages, which present antigens and activate the T-cells, and promote their differentiation into effector T-cells. Tumor size is known to negatively affect the activity of effector T-cells and NK cells. Similarly, presence of immunosuppressor cells in tumor microenvironment and exhaustion have negative effects on the activity of effector T-cells and NK cells. Finally, decreased infiltration of effector T-cells and NK cells in the tumor also leads to decreased anti-tumor immune response. In the flow diagram, all the major processes that control the anti-tumor immune response are presented as nodes. (+) indicates positive effect of the molecule/target on the node and (-) indicates inhibitory effect of the molecule/target on the node

Killing of tumor cells and eradication of tumors from the body by the immune system is illustrated in Fig. 3 with key events highlighted as nodes. Tumor size has been shown to be negatively associated with activation of immune response [27–30] and tumor cell cytotoxicity can be achieved by activated effector T-cells and natural killer (NK) cells [31, 32]. The levels of activated effector T-cells and NK cells in the tumor microenvironment (TME) is dependent on the ability of the cells to infiltrate the TME and decreased tumor infiltration of immune cells is a common mechanism of immune escape [33–35]. Activation of T-cells is dependent on antigen presentation and on phenotype of antigen presenting cells including dendritic cells (DCs) and macrophages [36–38]. Activity of effector T-cells and NK depends on levels of immune suppressor cells such as regulatory T-cells (Tregs) and myeloid-derived suppressor cells (MDSCs) in the TME. Similarly, immune cell exhaustion is known to negatively affect the activity of effector T-cells and NK cells. Immune checkpoints such as PD-1, CTLA-4, T cell immunoreceptor with Ig and ITIM domains (TIGIT), T cell immunoglobulin and mucin domain-containing protein 3 (Tim-3) and lymphocyte activation gene-3 (Lag-3) inhibit the activation of effector T-cells and NK cells and promote exhaustion.

As illustrated in Fig. 3, drugs can act at multiple nodes and activate the anti-tumor immune response. Drugs that have direct cytotoxic effects, such as chemotherapeutics, can positively influence the immune response by promoting antigen release and thereby modulating DC/macrophage phenotype and antigen presentation; and also, by reducing the concentration of immune-suppressor cells such as Tregs and MDSCs in the TME. Drugs such as selicrelumab can activate effector immune cells directly and indirectly by activating antigen presenting cells (APCs). Monoclonal antibodies against immune checkpoints can block inhibitory effects of checkpoints to reinvigorate exhausted immune cells, regulate APC phenotype (CTLA-4 and TIGIT blockers) and directly modulate antigen presentation (Tim-3 blockers).

**Fig. 4:**
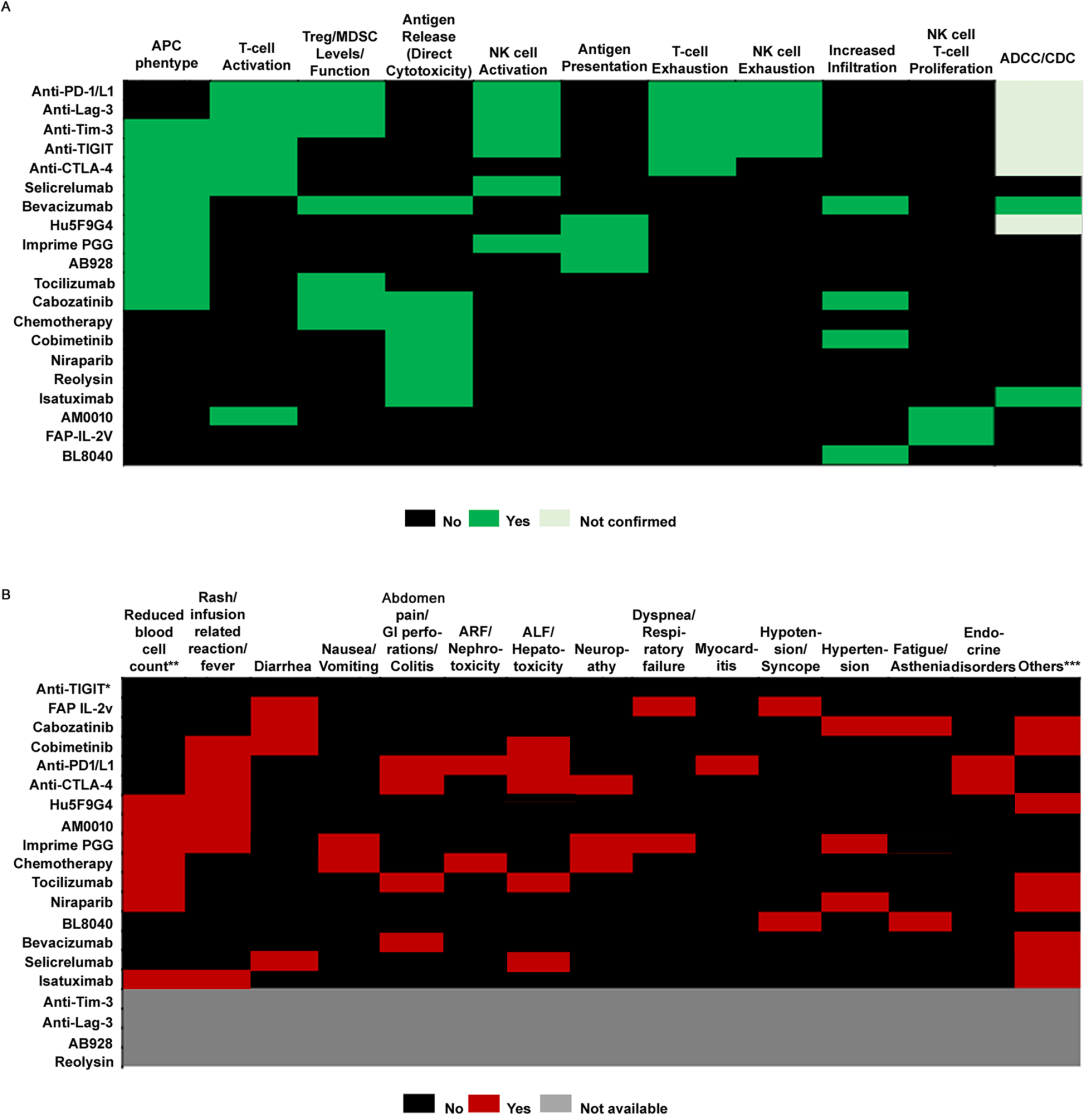
Heat map showing overlap of (**A**) Mechanism of action (**B**) Serious AEs and/or Grade 3 or above AEs for screened drugs (**A**) *chemotherapy has also been shown in some studies to downregulate PD-L1 and PD-L2 expression on DCs and induce cytotoxic activity of CTLs and NK cells, **B** * Early reports from clinical studies evaluating TIGIT did not report any dose limiting toxicities, except a case of grade 2 diarrhea. **Reduced blood cell count is used as a broad category of AEs and includes direct suppression of bone marrow generation of blood cells as well as indirect reductions in blood cell counts resulting in neutropenia, anemia, decreased lymphocyte count and thrombocytopenia. *** SAEs sorted in the ‘Others’ category are sometimes unique for the drug and cannot be combined as a single category. Early phase 1 studies for anti-Tim-3, anti-Lag-3, AB928 and Reolysin did not report serious adverse events but evidence from studies in larger cohort is not available and are represented accordingly (grey). Data includes rare events and may include AEs that are probably not related to study. ARF, acute renal failure; ALF, acute liver failure

On the other hand, multiple drugs can act on a single node and with overlapping mechanisms of action as shown in Fig. 4A. In the drugs screened for the final summary of efficacy, primary overlap was seen in their effects on APC phenotype/maturation, T-cell activation, levels/function of immune suppressor cells and tumor size/antigen release (Fig. 4A). Combination of chemotherapy and PD-1/PD-L1 blockers, which has been approved for treatment of metastatic non-small cell lung cancer (NSCLC) [39–42], had minimal overlap of mechanisms. Chemotherapy affected the tumor size, induced antigen release and reduced the levels of immune suppressor cells, while PD-1/PD-L1 blockers where shown to activate T-cells, NK-cells and inhibit function and maturation of immune suppressor cells (Fig. 4A). Similarly, bevacizumab additionally induced APC maturation and increased immune cell infiltration [43–45]. Ipilimumab, which stimulated APC phenotype and induced central activation of T-cells was a successful combination with PD-1 blockers [46–48]. Details from Fig. 4A can thus be used to deduce possibilities of synergism in combinations with complementary non-overlapping mechanisms, or additive effects in novel combinations with some degree of overlapping mechanisms.

While understanding the overlap of molecular mechanism of action could provide an overview of efficacy of the combination, combinations can fail to translate their success from preclinical to clinical studies. Data from clinical studies would therefore be critical in estimating the potential of the combination. Table 3 summarizes the available efficacy data (solid tumors) for the drugs selected in our final stage of screening and scores the data based on availability. For drugs and class of drugs that are approved as monotherapy, only the key studies that are relevant for discussion are summarized. Isatuximab, which was studied mainly in heme cancers, received the lowest score on data availability, while drugs in early clinical development including FAP-IL-2 V, BL-8040, Hu5F9G4, AB928 and tocilizumab (approved in the non-oncology setting), received moderate scores.

**Table 3:** Efficacy outcomes reported for screened drugs

Compound	Efficacy as monotherapy or in combination with chemotherapy or targeted therapy	Efficacy in combination with PD-1/PD-L1 blockers	Clinical status	Clinical score
Ipilimumab	Monotherapy for unresected melanoma [Yervoy™ package insert]: ORR, 10.9 %; median OS, 10 months; HR for OS, 0.66	Combination with nivolumab [Yervoy™ package insert]: ORR, 36 %; median PFS, 5.1 months; HR for PFS, 0.82; median OS, 17.1 months; HR for OS, 0.79 Combination with nivolumab and platinum-doublet chemotherapy [Yervoy™ package insert]: ORR, 38 %; median PFS, 6.8 months; HR for PFS, 0.70: median OS, 14.1 months, HR for OS, 0.69	Phase 4 and post market studies	3
Tim-3 blockers	Efficacy data not available	Efficacy data not available	Multiple phase 2 studies	1
Lag-3 blockers	Efficacy data not available	Combination with Nivolumab for metastatic melanoma [49]: Sponsor reported through press release that the combination met the primary endpoint of PFS. Details have not been published at the time of reporting.	Multiple phase 2 and phase 2/3 studies	1
TIGIT blockers	Tiragolumab for solid tumors [50]: Stable disease in 4 of 24 patients	Tiragolumab with Atezolizumab [50]: ORR, 37 %; median PFS, 5.6 months	Multiple phase 3 studies	3
Chemotherapy	Carboplatin + nab-paclitaxel [51]: ORR, 33 %; DCR, 53 %; median PFS, 6.3 months; median OS, 12.1 months Carboplatin + sb-paclitaxel [51]: ORR, 25 %; DCR, 49 %; median PFS, 5.8 months; median OS, 11.2 months	Combination with Pembrolizumab, Carboplatin & paclitaxel/nab-paclitaxel [40]: ORR, 58 %; median PFS 6.4 months; HR for PFS, 0.56; median OS, 15.9 months; HR for OS, 0.64 Combination with Pembrolizumab, Platinum based drug & pemtrexed [39]: ORR, 48 %; DCR, 85 %; median PFS 8.8 months; HR for PFS, 0.52; median OS, not reached; HR for OS, 0.49 Combination with Atezolizumab, Carboplatin & nab-paclitaxel [52]: ORR, 49 %; DCR, 79 %; median PFS, 7.0 months; HR for PFS, 0.64; median OS, 18.6 months; HR for OS, 0.79	Phase 4 and multiple post market studies	3
Bevacizumab	Combination with docetaxel [53]: ORR, 36 %; DCR, 62 %; median PFS, 4.4 months; HR for PFS, 0.71; median OS, 13.1 months; HR for OS, 0.74 Combination with carboplatin & paclitaxel [54]: ORR, 42 %; DCR, 85 % Combination with pemetrexed and carboplatin [55]: ORR, 34 %; DCR, 66 %; median PFS, 6.0 months; HR for PFS, 0.83; median OS, 12.6 months; HR for OS, 1.0 Combination with erlotinib [56]: ORR, 69 %; DCR, 99 %; median PFS, 16.0 months; HR for PFS, 0.54	Combination with Atezolizumab & chemotherapy [43]: ORR, 64 %; DCR, 85 %; median PFS, 8.3 months; HR for PFS, 0.62; median OS 19.2 months; HR for OS, 0.78	Multiple phase 3 studies	3
FAP-IL-2 V	Monotherapy* [57]: ORR, 7 %; DCR, 45 %	Data not available	Multiple phase 3 studies	2
Cobimetinib	Combination with vemurafenib (melanoma) [58]: ORR, 67 %; DCR, 87 %; median PFS, 11.3 months; HR for death or disease progression, 0.60	Combination with atezolizumab (CRC) [59]: ORR, 3 %; DCR, 26 %; median PFS, 1.9 months; median OS, 8.9 months;	Multiple phase 3 studies	3
Imprime PGG	Combination with cetuximab and chemotherapy [60]: ORR, 37 %; DCR, 85 %; median OS, 10.3 months; HR for OS, 1.14 Combination with bevacizumab and chemotherapy [61]: ORR, 60 %; DCR, 94 %; median PFS, 11.6 months; HR for PFS, 1.31; median OS, 16.1 months; HR for OS, 0.75	Combination with pembrolizumab (TNBC) [62]: ORR, 16 %; DCR, 55 %; median OS, 13.7 months	Phase 1 and Phase 2 studies	3
AM0010	Monotherapy (solid tumors) [63]: ORR, 21 % Monotherapy (RCC) [63]: ORR﻿, 27 %	Combination with pembrolizumab or nivolumab [64]: NSCLC subset: ORR, 43 %; DCR, 82 %; median PFS, 9.4 months; median OS, 24.1 months Melanoma subset: ORR, 10 %; DCR, 52 %; median PFS, 2.2 months; median OS, 16.7 months RCC subset: ORR, 40 %; DCR, 86 %; median PFS, 12.5 months; median OS, not reached	Phase 2 study	3
BL8040	Data not available	Combination with pembrolizumab and chemotherapy (PDAC) [65]: ORR, 3 %; DCR, 34 %; median OS, 3.3 months	Multiple phase 1/2 studies	2
Selicrelumab	Monotherapy (solid tumors, dose escalation study) [66]: ORR, 14 %; DCR, 38 % Combination with gemcitabine (PDAC) [67]: ORR, 19 %; DCR, 71 %; median PFS, 5.2 months; median OS, 8.4 months Combination with tremelimumab (melanoma) [68]: ORR 27 %; median PFS, 3.2 months; median OS 23.6 months Combination with cisplatin and pemetrexed (mesothelioma) [69]: ORR, 40 %; DCR, 93 %; median PFS, 6.3 months; median OS, 16.5 months Combination with carboplatin and paclitaxel (solid tumors) [70]: ORR, 20 %; DCR, 60 %	Data not available	Multiple phase 1 studies	3
Reolysin	Combination with carboplatin and paclitaxel: NSCLC [71]: ORR, 31 %; DCR, 89 %; median PFS, 4.0 months; median OS, 13.1 months Melanoma [72]: ORR, 21 %; DCR, 85 %; median PFS, 5.2 months; median OS, 10.9 months PDAC [73]: ORR, 20 %; DCR, 74 %; median PFS, 4.9 months; median OS, 7.3 months Ovarian cancer [74]: ORR, 17 %; DCR, 52 %; median PFS, 4.4 months; median OS, 12.6 months	Combination with pembrolizumab and chemotherapy (MAP) [75, 76]: DCR, 30 % in efficacy evaluable patients	Multiple phase 2 and phase 3 studies	3
Hu5F9G4	Monotherapy (solid tumors) [77]: Dose finding study. 2 patients (ovarian and fallopian tube cancers) treated with weekly maintenance doses at 20 mg/kg had confirmed partial responses with time to progression 5.2 months and 9.2 months respectively. Combination with cetuximab (CRC)﻿ [78]: ORR, 6.7 %; median PFS, 3.6 months; median OS, 10.1 months in KRASwt patients. SD, 45 %; median PFS, 1.9 months; median OS, 10.4 months in KRASm patients.	Data not available	Multiple phase 1 studies	2
Cabozatinib	Monotherapy [79]: ORR, 10 %; DCR, 38 %; median PFS, 2.4 months; median OS, 7.7 months Monotherapy or plus erlotinib [80]: ORR, 11 %; DCR, 61 %; median PFS, 4.3 months; median OS, 9.2 months; HR for OS 0.51. Combination arm: ORR, 3 %; DCR, 49 %; median PFS, 4.7 months; median OS, 13.3 months	Combination with nivolumab (RCC) [81]: ORR, 56 %; median PFS, 16.6 months; HR for PFS, 0.51; median OS, not reached; HR for OS, 0.60	Multiple phase 3 studies	3
AB928	Combination with modified FOLFOX-6 (CRC) [82]: DCR 76 %	Combination with chemotherapy or PD-1 blocker AB122 (solid tumors) [83]: DCR, 43 % (phase 1 study)	Multiple phase 1 studies	2
Niraparib	Monotherapy PRIMA (OC) [84]: median PFS, 13.8 months; HR for PFS, 0.62; HR for OS, 0.70 QUADRA (OC) [85]: ORR, 10 %; median PFS, 5.5 months; median OS, 17.2 months CRPC [86]: ORR, 38 %	Combination with pembrolizumab (TNBC) [87]: ORR, 21 %; DCR, 49 %; median PFS, 8.3 months OC [88]: ORR, 18 %; DCR, 65 %; median PFS, 3.4 months	Multiple phase 3 studies	3
Tocilizumab	Combination with chemotherapy and interferon-α2b (Ovarian Cancer) [89]: ORR, 48 %; DCR, 74 %; median OS, 54 weeks	Data not available	Multiple phase 1/2 studies	2
Isatuximab	Isatuximab is mainly studied in Heme cancers (multiple myeloma). Data in solid tumors is not available.	Combination with atezolizumab (CRC) [90]: No response was seen. SD, 20 %; DCR, 6.7 %; median PFS, 1.4 months; median OS, 5.1 months. Combination did not show superior efficacy over control treatment.	Multiple phase 3 studies	1

Note: Solid tumors mainly NSCLC are preferentially reported over others. Unless otherwise indicated, data shown in the table are from studies in lung cancer patients. Data presented in the table represents the modified intent to treat population (mITT) where reported and is extracted from the posters of conferences and peer reviewed publications. Details on the Clinical Score in the Table 2: 3 is better, 1 is lower

Abbreviations: *MAP* metastatic adenocarcinoma of pancreas, *PDAC* pancreatic ductal adenocarcinoma, *CRC* colorectal cancer, *TNBC* triple negative breast cancer, *OC* ovarian cancer, *CRPC* castration-resistant prostate cancer

#### Safety: overlap and potential for severe adverse events

Another important factor to be weighed while designing combination therapies is the potential for increased prevalence of severe AEs, which can lead to cessation of therapy or to a fatal outcome. Immunotherapy is considered to have a comparatively mild to moderate safety profile and AEs are mostly managed with corticosteroids [91–93]. However, severe dose-limiting immune-related AEs such as hepatitis and myocarditis, colitis, and endocrine disorders, are reported in some patients [91, 92, 94]. More importantly, adverse events of special interest were found to be more commonly reported in responding patients compared to non-responders [95]. Combination of PD-1 and CTLA-4 blockers has been associated with increased incidence of adverse events [46, 47, 96, 97] and with precipitation of severe myocarditis in some patients [98, 99]. Furthermore, the incidence of dose-limiting grade 3–4 AEs is also higher with combination immunotherapy; which was over 50 % in melanoma patients, over 30 %-50 % in lung, prostate and esophageal cancer patients and 14 % in patients with unresectable sarcoma with combination of ipilimumab and nivolumab [100]. Interestingly, incidence of grade 3–4 AEs mirrored the response to therapy and the cancers that were most responsive to the combination had highest incidence [100]. While on one hand, the incidence of all AEs and grade 3–4 AEs provides an overall idea of the safety profile, it cannot clearly identify a risky/unsafe combination. It is possible that the safety profile of drugs used in a combination does not overlap resulting in an overall increase incidence of AEs but without precipitation of serious adverse events. On the other hand, drugs may have manageable safety profile as monotherapy but could precipitate serious AE in susceptible patients when used in combination.

Understanding the safety profile of the drugs used in a combination and the overlap of AEs is essential in designing safe combination therapies. Supplementary Table S4 lists the commonly reported AEs and serious/dose-limiting, grade 3 or above AEs for the drugs selected in our final stage of screening. Figure 4B visualizes serious/dose-limiting, grade 3 or above AEs from Supplementary Table S4 as a heat map and illustrates the possible overlap of AEs. The set of AEs that were most commonly reported included rash, infusion site reaction and fever, followed by abnormalities in blood cell counts, liver abnormalities and gastrointestinal abnormalities (Fig. 4B). The heatmap of AEs presented in Fig. 4B also identifies the possible cases where serious AE can be precipitated. For example, dyspnea and respiratory failure might be expected to be severe in a combination with FAP-IL2v and Imprime PGG. Similarly, hypotension and syncope can be expected to be severe in a combination with FAP-IL2v and BL-8040 (Fig. 4B). While Supplementary Table S4 and Fig. 4B list the commonly seen AEs and illustrate their overlap, they do not capture the incidence or rate of AEs. Information from the AE heatmap together with the incidence of AE can help in identifying potential common serious adverse events.

### Strengths and limitations

This review presents a unique approach of identifying potential combinations of high interest for clinical development. The literature was extensively screened for various key pieces of information on mechanism of action, which provides an initial indication of whether the combination has potential for success. The overlap of mechanisms may also provide a possible indication on whether the combination could have additive or synergistic activity. The safety data curated from the literature helps our understanding of the overall AE profile and AE overlap of combinations help in identifying serious AEs in need of careful safety monitoring. Combined analysis of mechanism and safety overlaps for a combination can thus help in anticipating the likelihood of success. For example, looking at the overlaps in the mechanisms of action and safety profiles of anti-VEGF drugs and PD-(L)1 blockers, we see a complementary overlap in mechanisms but no overlap in serious AEs, which could explain the success of bevacizumab plus atezolizumab and cabozatinib plus nivolumab combinations in the clinic. Further, the efficacy data included in the review for the drugs in early stages of clinical development could help rescue drugs with marginal monotherapy efficacy but with promising combination outcomes, as suggested by this analysis and vice versa. Lastly, the methods and ranking protocols used in this review could also be useful in developing combinations based on additional targets introduced in the future.

Our review is not a one size fits all approach but can provide a few examples that assist with asset prioritization, ranking and decision making. One of the main limitations of this review is that it is not a true systematic review, and as such selection bias with respect to the drugs that are included, and literature related to the included drugs is not ruled out. For example, we limited the selection of drugs to a few representatives of each mode of action category to favor diversity of drug mechanisms. Radiation therapy, which is an integral part of cancer treatment could not be included in the review because the scope was limited to pharmacological therapy [101]. Similarly, cell-based therapies such as cancer vaccines, NK cells and chimeric antigen receptor T cells (CAR-T cells) and antibody therapies such as bispecific antibodies could not be included due to limited applications. The ranking of the drugs was mainly based on the availability of clinical data and drugs that were excluded due to lack of clinical data could show promising clinical efficacy in future studies. While strategies for prioritizing drugs in preclinical and early clinical stages was out of scope for this review, we think drugs with promising safety in preclinical studies should be given more weight over efficacy during the development of ranking strategy. Lastly, patient selection, biomarkers, disease/patient prognostic factors, dosage of combinations and sequence of administration that are known to be of significant influence on the success of combination cancer immunotherapies were also out of the scope of the review and were not discussed. This review should be therefore cautiously interpreted and applied with due consideration of the limitations.

## Summary

Specific strategies for therapies towards a large number of targets with modern cancer immunotherapy combinations that broadly benefit a larger number of patients with cancer must be brought forward. The analysis conducted herein aims to better understand particular characteristics of potential drugs to co-target in new combinations. We aim to provide clinically relevant insights and quantitative pharmacology-based ranking as a tool to improve combination testing. Our goal is to raise awareness of the multitude of issues impacting combination selection and development to draw attention to the need for further fine-tuned methodologies for more optimal selection for both current and future clinical trials. Our review encourages future efforts of this sort to rank combinations of highest interest as to how they may interact when given together, with a certain consideration for when to move forward, and potential items of caution. While our focus is on patients with metastatic cancers, there is also a paradigm shift and recent focus to test combination immunotherapies against earlier stages of cancer, and similar strategies described herein can also be considered for combination trials in the neoadjuvant and adjuvant setting [4, 102]. Expansion of the quantitative pharmacology based ranking approach herein may be addressed for new targets, lines of therapies, and biomarker selected populations. Moving forward, creative umbrella, basket, broad, and flexible platform trial designs across multiple disease areas that adapt and scale to emerging safety and efficacy findings will be key to testing.

## Conclusion

In conclusion, our review highlights the need for strategies to prioritize and rank the potential leads for combination immunotherapy and proposes quantitative pharmacology-based ranking as an approach. Comprehensive ranking based on fundamental molecular and cellular pharmacological foundations and relevant mechanisms of action to hit multiple targets may at least provide a partial solution to the complexity challenge by better predicting optimal strategies.

## Supplementary Information

**Additional file 1: Supplementary Table S1.** Immunotherapy combinations approved for cancer treatment. **Supplementary Table S2.** Ranking of drugs based on knowledge of MoA, relevance to immunotherapy and importance of indication. Details on the scoring scale is provided in the Table 2. Higher scores are better. **Supplementary Table S3.** Details of drugs selected in the final phase of screening. **Supplementary Table S4.** Safety of final phase drugs reported in NSCLC, SCLC, or melanoma.

## Data Availability

All data generated in the study has been included in the manuscript and accompanying supplementary information.

## Abbreviations

PD-1: Programmed cell death protein 1
CTLA-4: Cytotoxic T-lymphocyte antigen 4
VEGF: Vascular endothelial growth factor
TMB: Tumor mutational burden
ORR: Objective response rate
CR: Complete response
PR: Partial response
DCR: Disease control rate
PFS: Progression free survival
OS: Overall survival
MOA: Mechanism of action
AE: Adverse event
NK cells: Natural killer cells
TME: Tumor microenvironment
DCs: Dendritic cells
Tregs: Regulatory T-cells
MDSCs: Myeloid-derived suppressor cells
TIGIT: T cell immunoreceptor with Ig and ITIM domains
Tim-3: T cell immunoglobulin and mucin domain-containing protein 3
Lag-3: Lymphocyte activation gene-3
APCs: Antigen presenting cells
CAR-T cells: Chimeric antigen receptor T cells
mITT: Modified intent to treat population
NSCLC: Non-small cell lung cancer
MAP: Metastatic adenocarcinoma of pancreas
PDAC: Pancreatic ductal adenocarcinoma
CRC: Colorectal cancer
TNBC: Triple negative breast cancer
OC: Ovarian cancer
CRPC: Castration-resistant prostate cancer
