# Supplementary material for: Pharmacology-based ranking of anti-cancer drugs to guide clinical development of cancer immunotherapy combinations
Source: J Exp Clin Cancer Res. 2021 Oct 1;40:311. doi: 10.1186/s13046-021-02111-5 (PMC8485537; doi:10.1186/s13046-021-02111-5)
Supplement: Supplementary file 1 — Additional file 1: Supplementary Table S1. Immunotherapy combinations approved for cancer treatment. Supplementary Table S2. Ranking of drugs based on knowledge of MoA, relevance to immunotherapy and importance of indication. Details on the scoring scale is provided in the Table 2. Higher scores are better. Supplementary Table S3. Details of drugs selected in the final phase of screening. Supplementary Table S4. Safety of final phase drugs reported in NSCLC, SCLC, or melanoma. [file 13046_2021_2111_MOESM1_ESM.docx]

# **Supplementary Information**

**Supplementary Table S1**. Immunotherapy combinations approved for cancer treatment

| Combination | Marketed by | Indication |
| --- | --- | --- |
| Nivolumab plus Ipilimumab | Bristol-Myers Squibb | Metastatic melanoma  Metastatic NSCLC with no EGFR or ALK aberrations  Untreated advanced renal cell carcinoma  Metastatic colorectal carcinoma  Metastatic hepatocellular carcinoma  Metastatic mesothelioma |
| Nivolumab plus Ipilimumab and 2-cycles of platinum doublet chemotherapy | Bristol-Myers Squibb | Metastatic non-squamous NSCLC with no EGFR or ALK aberrations |
| Nivolumab plus cabozatinib | Bristol-Myers Squibb | Advanced renal cell carcinoma |
| Pembrolizumab plus Chemotherapy | Merck | Metastatic non-squamous NSCLC with no EGFR or ALK aberrations  Metastatic squamous NSCLC |
| Pembrolizumab plus Axitinib | Merck | Advanced renal cell carcinoma |
| Atezolimumab plus Bevacizumab and Chemotherapy | Roche/Genentech | Metastatic non-squamous NSCLC |
| Atezolizumab plus Chemotherapy | Roche/Genentech | Metastatic non-squamous NSCLC  Extensive-stage SCLC  Unresectable, advanced TNBC |
| Atezolizumab plus Bevacizumab | Roche/Genentech | Metastatic hepatocellular carcinoma |
| Atezolizumab plus Cobimetenib and Vemurafenib | Roche/Genentech | BrafV600E^+^ metastatic melanoma |

**Supplementary Table S2**. Ranking of drugs based on knowledge of MoA, relevance to immunotherapy and importance of indication. Details on the scoring scale is provided in the **Table 2**. Higher scores are better.

| Compound | Molecular Target | Relevance of Indication | Effects on Immune response | Knowledge of MoA | Overall Score |
| --- | --- | --- | --- | --- | --- |
| PD-1/PD-L1 blockers | PD-1/PD-L1 | 3 | 3 | 3 | 9 |
| CTLA-4 blockers | CTLA-4 | 2 | 3 | 3 | 8 |
| Tim-3 blockers | Tim-3 | 2 | 3 | 3 | 8 |
| LAG-3 blockers | LAG-3 | 2 | 3 | 3 | 8 |
| TIGIT blockers | TIGIT | 2 | 3 | 3 | 8 |
| FAP-IL2v | IL-2 receptor | 2 | 2 | 3 | 7 |
| Chemotherapy | Cancer cell DNA | 3 | 2 | 3 | 8 |
| Cabozantinib | RTK | 2 | 2 | 3 | 7 |
| Bevacizumab | VEGF | 3 | 2 | 3 | 8 |
| Cobimetinib | MEK | 3 | 2 | 3 | 8 |
| BL8040 | CXCR4 | 3 | 2 | 3 | 8 |
| Niraparib | PARP | 3 | 2 | 3 | 8 |
| Selicrelumab | CD40 (agonist) | 2 | 3 | 3 | 8 |
| Hu5F9-G4 | CD47 | 2 | 3 | 3 | 8 |
| AB928 | Adenosine receptor | 2 | 2 | 3 | 7 |
| AM0010 | IL-10 receptors (agonist) | 2 | 3 | 3 | 7 |
| Reolysin | Isolate of the unmodified REOVIRUS (Respiratory Enteric Orphan Virus) | 2 | 2 | 3 | 7 |
| Tocilizumab | IL-6 | 2 | 3 | 3 | 8 |
| Imprime PGG | TLRs | 2 | 2 | 3 | 7 |
| Isatuximab | CD38 (agonist) | 2 | 2 | 3 | 7 |
| K01401-020 | VISTA | 2 | 2 | 3 | 7 |
| GB1275 | CD11b | 2 | 2 | 3 | 7 |
| CMP-001 | TLR9 | 2 | 1 | 3 | 6 |
| CPI444 | Adenosine receptors | 1 | 2 | 3 | 6 |
| LOAd703 | Oncolytic adenovirus | 1 | 2 | 3 | 6 |
| CDX301 | FLT3 receptors | 1 | 2 | 3 | 6 |
| Idasanutlin | MDM2 | 1 | 2 | 3 | 6 |
| CCX872 | CCR2 | 1 | 1 | 3 | 5 |
| PEGPH 20 | PEGylated human recombinant hyaluronidase | 1 | 1 | 3 | 5 |
| Venetoclax | Bcl2 | 1 | 1 | 3 | 5 |
| DV-281 | TLR-9 agonist | 1 | 1 | 3 | 5 |
| Linagliptin | DPP-4 | 1 | 2 | 2 | 5 |
| Anti-IL8 | IL-8 | 1 | 1 | 3 | 5 |
| Canakinumab | IL-1b | 1 | 1 | 3 | 5 |

**Supplementary Table S3**. Details of drugs selected in the final phase of screening

| Compound | Indications & Setting | Phase of studies | Effects on immune response | References |
| --- | --- | --- | --- | --- |
| PD-1/PD-L1 blockers | Multiple indications, both as adjuvant and metastatic | Ph4 and post market studies | Blocks PD-1/PD-L1 mediated inhibition of effector immune cells. Peripheral activation of effector T-cells and NK cells. Inhibits PD-1/PD-L1 mediated TReg differentiation and exhaustion of T-cells and NK cells | (1,2) |
| CTLA-4 blockers | Cutaneous melanoma both as adjuvant and metastatic  In combination with PD-1 blockers for multiple indications including lung cancer | Ph4 and post market studies | Blocks CTLA-4 mediated inhibition of effector immune cells. Peripheral activation of effector T-cells. Blocks CTLA-4 mediated induction of tolerogenic APCs and exhaustion of T-cells. | (1,2) |
| Tim-3 blockers | Currently under clinical investigation | Multiple Ph3 studies | Block Tim-3 mediated inhibition of effector immune cells. Peripheral activation of effector T-cells and NK cells. Inhibits accumulation of TRegs and MDSCs. Blocks Tim-3 mediated exhaustion of T-cells and NK cells. | (1,2) |
| Lag-3 blockers | Currently under clinical investigation | Multiple Ph 3 studies | Block LAG-3 mediated inhibition of effector immune cells. Direct activation of effector T-cells and NK cells. Inhibits TReg functions. Blocks Lag-3 mediated exhaustion of T-cells and NK cells. | (1,2) |
| TIGIT blockers | Currently under clinical investigation | Multiple Ph3 studies | Block TIGIT mediated inhibition of effector immune cells. Direct activation of effector T-cells and NK cells. Blocks TIGIT mediated exhaustion of T-cells and NK cells. | (1,2) |
| Chemotherapy | Multiple indications, both as adjuvant and metastatic. Approved in combination with PD-1/L1 blockers for NSCLC, and TNMBC | Ph4 and post market studies | Release of antigens and Danger Associated Molecular Patterns through cytotoxic action on cancer cells; Depletion of Tregs; Downregulation of PD-L1/L2 expression | (3-5) |
| Bevacizumab | Multiple indications as monotherapy. Approved in combination with Atezolizumab (PD-L1 blocker) for metastatic hepatocellular carcinoma. | Multiple Ph3 studies | Anti-VEGF antibody; Facilitates infiltration of CD8 T cells, NK cells into the TME; accelerates maturation of DCs | (6-8) |
| FAP-IL-2V | Currently under investigation | Multiple Ph3 studies | Preferentially targets tumor and selectively binds to IL2Rβγ receptors thereby avoiding expansion of Tregs | (9,10) |
| Cobimetinib | In combination with vemurafenib and/or atezolizumab for metastatic melanoma | Multiple Ph3 studies | MEK inhibitor; Prevents tumor cell proliferation and promotes infiltration of CTLs | (11) |
| Imprime PGG | Currently under investigation | Ph1 and Ph2 studies | Pathogen Associated Molecular Patterns; Stimulates cytotoxicity of innate immune cells (NK cells, neutrophils, macrophages); promotes transformation of macrophages from M2 to M1 | (12,13) |
| AM0010 | Currently under investigation | Ph2 study | Pegylated recombinant human IL-10, shown to stimulate proliferation and cytotoxicity of CTLs | (14,15) |
| BL8040 | Currently under investigation; FDA granted orphan drug designation for pancreatic cancer | Multiple Ph1/2 studies | Blocks CXCR4; Blockade of CXCR4-CXCL12 signaling facilitates infiltration of CD8 T cells from the tumor stroma into the tumor core | (16) |
| Selicrelumab | Currently under investigation | Multiple Ph1 studies | CD40 agonist, shown to recruit complement dependent cytotoxicity (CDC) and natural killer (NK) cells and facilitate tumor killing | (17,18) |
| Reolysin | Currently under investigation; FDA granted orphan drug designation for pancreatic cancer | Multiple Ph2 and Ph3 studies | Non-pathogenic immune-oncolytic virus that selectively kills RAS mutation positive tumor cells and releases antigens | (19,20) |
| Hu5F9G4 | Currently under investigation | Multiple Ph 1 studies | CD47 blocker, shown to induce tumor cell phagocytosis by macrophages and subsequent cross-presentation of tumor antigens | (21) |
| Cabozantinib | Approved for advanced renal cell carcinoma (RCC) and progressive metastatic medullary thyroid cancer | Multiple Ph3 studies | Receptor Tyrosine Kinase (RTK) inhibitor, shown to inhibit tumor growth as well as growth of immune suppressive MDSCs and Tregs | (22) |
| AB928 | Currently under investigation | Multiple Ph1 studies | Adenosine receptor antagonist (A_2A_R and A_2B_R), restores maturation and activation of dendritic cells and improves cytotoxicity of immune cells | (23,24) |
| Niraparib | Maintenance therapy and 2^nd^ line therapy for recurrent epithelial ovarian, fallopian and primary peritoneal cancers | Multiple Ph3 studies | PARP inhibitor, causes immunogenic cell death of cancer cells and release of neo-antigens | (25,26) |
| Tocilizumab | Currently under investigation for Cancer | Multiple Ph1/2 studies | IL-6 receptor blocker, shown to inhibit immunosuppressive actions of IL-6 | (27,28) |
| Isatuximab | Currently under investigation for Heme cancers | Multiple Ph3 studies | Anti-CD38 antibody, shown to induce target cell lysis though activation of ADCC, ADCP and CDC | (29,30) |

**Supplementary Table S4.** Safety of final phase drugs reported in NSCLC, SCLC, or melanoma

| Compound | Common AEs | Serious/Dose limiting/fatal AEs | References |
| --- | --- | --- | --- |
| PD-1/PD-L1 blockers | Fatigue, musculoskeletal pain, decreased appetite, diarrhea, nausea, abdominal pain, rash, cough, dyspnea, pyrexia and pruritus | Heptatotoxicity, Nephrotoxicity, endocrine disorders, pneumonitis, colitis, rash and infusion-related reaction | (31)  & package inserts of respective marketed drug |
| CTLA-4 blockers | Fatigue, diarrhea, pruritus, rash, and colitis. | Infusion related reactions, Colitis, hepatotoxicity, dermatitis, endocrine disorders and neuropathy | Yervoy package insert |
| Tim-3 blockers | Pruritus, rash maculo-popular, abdominal pain, amylase increase, infusion site reactions, arthralgia, ear pain, fatigue, nausea, allergic rhinitis | No dose limiting or ≥grade 3 toxicity in early studies | (32) |
| Lag-3 blockers | Studies ongoing. Data not available | Data not available | (33) |
| TIGIT blockers | Fatigue, pruritus, anemia, arthralgia, infusion related reaction, arthralgia, decreased appetite, dermatitis acneiform, diarrhea, nausea and rash | Grade 2 Anemia and Diarrhea were the only severe AEs seen after administration of anti-TIGIT antibodies. | (34) |
| Chemotherapy | Neurotoxicity, cardiotoxicity, nephrotoxicity, gastrointestinal (GI) complications, such as nausea, vomiting, constipation, and diarrhea, myelosuppression, alopecia, fatigue, myalgia/arthralgia, LFT elevation, peripheral edema, and decreased appetite | Cisplatin: Nephrotoxicity; Carboplatin: Myelosuppression Oxaliplatin: Neurotoxicity Others: Nausea, vomiting, | (35) |
| Bevacizumab | GI perforation, hemorrhage, fistulae, wound healing complications, arterial & venous thromboembolic events (ATE; VTE), hypertension, Posterior reversible encephalopathy syndrome, nephrotic syndrome, proteinuria and congestive heart failure | GI perforation, Fistulae, hemorrhage and ATEs | Avastin package insert |
| FAP-IL-2V | Pyrexia, infusion-related reaction (IRR), nausea, fatigue, increased AST, decreased appetite, diarrhea, increased ALT, chills, vomiting, asthenia, peripheral edema, rash, pruritus, increased blood bilirubin, hypotension, and dyspnea | Hypotension, diarrhea and respiratory failure | (36) |
| Cobimetinib | Diarrhea, rash, fatigue, pyrexia, edema, nausea, and vomiting | Diarrhea, rash, hepatotoxicity and central serous retinopathy | (37) & Cotellic™ package insert |
| Imprime PGG | Neutropenia, thrombocytopenia, anemia, leukopenia, nausea, constipation, vomiting, infusion site reaction, abdominal pain upper, dyspnea, polyneuropathy, stomatitis, pyrexia, fatigue, and chills | Neutropenia, thrombocytopenia, nausea, vomiting, dyspnea, polyneuropathy, hypertension and fever | (38) |
| AM0010 | Anemia, Leukopenia, thrombocytopenia, fatigue, injection site reactions, abnormal liver function test, fatigue and fever | Anemia, thrombocytopenia and rash | (39) |
| BL8040 | Erythema, injection site edema, flushing, pruritus, hot flush, nausea, urticaria, headache, asthenia, chills, dizziness, parasthesia, hyperhidrosis, rash macular, pallor, vomiting, injection site hematoma, injection site induration and edema peripheral | Asthenia and syncope | (16) |
| Selicrelumab | Cytokine release syndrome, fever, rigors, chills, headache or back pain and abnormal liver function test | Diarrhea, elevated ALT and AST, and GI hemorrhage | (40,41) |
| Reolysin | Neutropenia, lymphopenia and hyponatremia (early data) | Data not available | (42) |
| Hu5F9G4 | Anemia, thrombocytopenia, decreased lymphocyte count, arthralgia, headache, fatigue, hyperbilirubinemia, pyrexia, chills, infusion related reaction, hemagglutination, and nausea | Anemia, decreased lymphocyte count, hemagglutination and infusion related reaction | (43) |
| Cabozatinib | Diarrhea, hypertension, fatigue, nausea, vomiting, decreased appetite, constipation, weight loss and palmar–plantar erythrodysesthesia syndrome | Diarrhea, hypertension, palmar–plantar erythrodysesthesia syndrome, and fatigue | (44) & Cabometyx™ package insert |
| AB928 | Nausea, abdominal pain, abdominal distension, headache, dizziness, constipation, palpitations and hot flush (early data) | Data not available | (23) |
| Niraparib | Myelosuppression, nausea, constipation, vomiting, hypertension, insomnia, decreased appetite, headache and dizziness | Myelosuppression, hypertension and embryo-fetal toxicity | Zejula™ package insert |
| Tocilizumab | Decreased neutrophil and platelet count, infections and infestations, abnormal liver function tests/hepatotoxicity, hypertension and headache | Serious infection, neutropenia and thrombocytopenia, hepatotoxicity, GI perforations | Actemra™ package insert |
| Isatuximab | Infusion related reactions, upper respiratory tract infection, neutropenia, nausea, diarrhea, vomiting and dyspnea | Infusion related reactions, febrile neutropenia and pneumonia | Sarclisa™ package insert |

**References**

1. Bhandaru M, Rotte A. Monoclonal Antibodies for the Treatment of Melanoma: Present and Future Strategies. Methods Mol Biol **2019**;1904:83-108 doi 10.1007/978-1-4939-8958-4_4.

2. Rotte A, Jin JY, Lemaire V. Mechanistic overview of immune checkpoints to support the rational design of their combinations in cancer immunotherapy. Ann Oncol **2018**;29(1):71-83 doi 10.1093/annonc/mdx686.

3. Kelland L. The resurgence of platinum-based cancer chemotherapy. Nat Rev Cancer **2007**;7(8):573-84 doi 10.1038/nrc2167.

4. Ramakrishnan R, Gabrilovich DI. Mechanism of synergistic effect of chemotherapy and immunotherapy of cancer. Cancer Immunol Immunother **2011**;60(3):419-23 doi 10.1007/s00262-010-0930-1.

5. Ramakrishnan R, Huang C, Cho HI, Lloyd M, Johnson J, Ren X*, et al.* Autophagy induced by conventional chemotherapy mediates tumor cell sensitivity to immunotherapy. Cancer Res **2012**;72(21):5483-93 doi 10.1158/0008-5472.CAN-12-2236.

6. Cohen MH, Gootenberg J, Keegan P, Pazdur R. FDA drug approval summary: bevacizumab (Avastin) plus Carboplatin and Paclitaxel as first-line treatment of advanced/metastatic recurrent nonsquamous non-small cell lung cancer. Oncologist **2007**;12(6):713-8 doi 10.1634/theoncologist.12-6-713.

7. Terme M, Tartour E, Taieb J. VEGFA/VEGFR2-targeted therapies prevent the VEGFA-induced proliferation of regulatory T cells in cancer. Oncoimmunology **2013**;2(8):e25156 doi 10.4161/onci.25156.

8. Terme M, Pernot S, Marcheteau E, Sandoval F, Benhamouda N, Colussi O*, et al.* VEGFA-VEGFR pathway blockade inhibits tumor-induced regulatory T-cell proliferation in colorectal cancer. Cancer Res **2013**;73(2):539-49 doi 10.1158/0008-5472.CAN-12-2325.

9. Mortara L, Balza E, Bruno A, Poggi A, Orecchia P, Carnemolla B. Anti-cancer Therapies Employing IL-2 Cytokine Tumor Targeting: Contribution of Innate, Adaptive and Immunosuppressive Cells in the Anti-tumor Efficacy. Front Immunol **2018**;9:2905 doi 10.3389/fimmu.2018.02905.

10. Liu R, Li H, Liu L, Yu J, Ren X. Fibroblast activation protein: A potential therapeutic target in cancer. Cancer Biol Ther **2012**;13(3):123-9 doi 10.4161/cbt.13.3.18696.

11. Ebert PJR, Cheung J, Yang Y, McNamara E, Hong R, Moskalenko M*, et al.* MAP Kinase Inhibition Promotes T Cell and Anti-tumor Activity in Combination with PD-L1 Checkpoint Blockade. Immunity **2016**;44(3):609-21 doi 10.1016/j.immuni.2016.01.024.

12. Chan AS, Jonas AB, Qiu X, Ottoson NR, Walsh RM, Gorden KB*, et al.* Imprime PGG-Mediated Anti-Cancer Immune Activation Requires Immune Complex Formation. PLoS One **2016**;11(11):e0165909 doi 10.1371/journal.pone.0165909.

13. Bose N, Ottoson NR, Qiu X, Harrison B, Lowe JR, Uhlik MT*, et al.* Immune Pharmacodynamic Responses of the Novel Cancer Immunotherapeutic Imprime PGG in Healthy Volunteers. J Immunol **2019**;202(10):2945-56 doi 10.4049/jimmunol.1801533.

14. Chan IH, Wu V, Bilardello M, Mar E, Oft M, Van Vlasselaer P*, et al.* The Potentiation of IFN-gamma and Induction of Cytotoxic Proteins by Pegylated IL-10 in Human CD8 T Cells. J Interferon Cytokine Res **2015**;35(12):948-55 doi 10.1089/jir.2014.0221.

15. Chan IH, Wu V, McCauley S, Grimm EA, Mumm JB. IL-10: Expanding the Immune Oncology Horizon. Receptors Clin Investig **2015**;2(4).

16. Abraham M, Pereg Y, Bulvik B, Klein S, Mishalian I, Wald H*, et al.* Single Dose of the CXCR4 Antagonist BL-8040 Induces Rapid Mobilization for the Collection of Human CD34(+) Cells in Healthy Volunteers. Clin Cancer Res **2017**;23(22):6790-801 doi 10.1158/1078-0432.CCR-16-2919.

17. Vonderheide RH, Flaherty KT, Khalil M, Stumacher MS, Bajor DL, Hutnick NA*, et al.* Clinical activity and immune modulation in cancer patients treated with CP-870,893, a novel CD40 agonist monoclonal antibody. J Clin Oncol **2007**;25(7):876-83 doi 10.1200/JCO.2006.08.3311.

18. Vonderheide RH, Burg JM, Mick R, Trosko JA, Li D, Shaik MN*, et al.* Phase I study of the CD40 agonist antibody CP-870,893 combined with carboplatin and paclitaxel in patients with advanced solid tumors. Oncoimmunology **2013**;2(1):e23033 doi 10.4161/onci.23033.

19. Mahalingam D, Patel S, Nuovo G, Gill G, Selvaggi G, Coffey M*, et al.* The combination of intravenous Reolysin and gemcitabine induces reovirus replication and endoplasmic reticular stress in a patient with KRAS-activated pancreatic cancer. BMC Cancer **2015**;15:513 doi 10.1186/s12885-015-1518-0.

20. Chakrabarty R, Tran H, Selvaggi G, Hagerman A, Thompson B, Coffey M. The oncolytic virus, pelareorep, as a novel anticancer agent: a review. Invest New Drugs **2015**;33(3):761-74 doi 10.1007/s10637-015-0216-8.

21. Liu X, Pu Y, Cron K, Deng L, Kline J, Frazier WA*, et al.* CD47 blockade triggers T cell-mediated destruction of immunogenic tumors. Nat Med **2015**;21(10):1209-15 doi 10.1038/nm.3931.

22. Deeks ED. Cabozantinib: A Review in Advanced Hepatocellular Carcinoma. Target Oncol **2019**;14(1):107-13 doi 10.1007/s11523-019-00622-y.

23. Seitz L, Jin L, Leleti M, Ashok D, Jeffrey J, Rieger A*, et al.* Safety, tolerability, and pharmacology of AB928, a novel dual adenosine receptor antagonist, in a randomized, phase 1 study in healthy volunteers. Invest New Drugs **2019**;37(4):711-21 doi 10.1007/s10637-018-0706-6.

24. Young A, Mittal D, Stagg J, Smyth MJ. Targeting cancer-derived adenosine: new therapeutic approaches. Cancer Discov **2014**;4(8):879-88 doi 10.1158/2159-8290.CD-14-0341.

25. Wang Z, Sun K, Xiao Y, Feng B, Mikule K, Ma X*, et al.* Niraparib activates interferon signaling and potentiates anti-PD-1 antibody efficacy in tumor models. Sci Rep **2019**;9(1):1853 doi 10.1038/s41598-019-38534-6.

26. Dziadkowiec KN, Gasiorowska E, Nowak-Markwitz E, Jankowska A. PARP inhibitors: review of mechanisms of action and BRCA1/2 mutation targeting. Prz Menopauzalny **2016**;15(4):215-9 doi 10.5114/pm.2016.65667.

27. Chang Q, Daly L, Bromberg J. The IL-6 feed-forward loop: a driver of tumorigenesis. Semin Immunol **2014**;26(1):48-53 doi 10.1016/j.smim.2014.01.007.

28. Dijkgraaf EM, Santegoets SJ, Reyners AK, Goedemans R, Wouters MC, Kenter GG*, et al.* A phase I trial combining carboplatin/doxorubicin with tocilizumab, an anti-IL-6R monoclonal antibody, and interferon-alpha2b in patients with recurrent epithelial ovarian cancer. Ann Oncol **2015**;26(10):2141-9 doi 10.1093/annonc/mdv309.

29. Moreno L, Perez C, Zabaleta A, Manrique I, Alignani D, Ajona D*, et al.* The Mechanism of Action of the Anti-CD38 Monoclonal Antibody Isatuximab in Multiple Myeloma. Clin Cancer Res **2019**;25(10):3176-87 doi 10.1158/1078-0432.CCR-18-1597.

30. van de Donk NW, Janmaat ML, Mutis T, Lammerts van Bueren JJ, Ahmadi T, Sasser AK*, et al.* Monoclonal antibodies targeting CD38 in hematological malignancies and beyond. Immunol Rev **2016**;270(1):95-112 doi 10.1111/imr.12389.

31. Sun L, Zhang L, Yu J, Zhang Y, Pang X, Ma C*, et al.* Clinical efficacy and safety of anti-PD-1/PD-L1 inhibitors for the treatment of advanced or metastatic cancer: a systematic review and meta-analysis. Sci Rep **2020**;10(1):2083 doi 10.1038/s41598-020-58674-4.

32. Harding JJ, Patnaik A, Moreno V, Stein M, Jankowska AM, de Mendizabal NV*, et al.* A phase Ia/Ib study of an anti-TIM-3 antibody (LY3321367) monotherapy or in combination with an anti-PD-L1 antibody (LY3300054): Interim safety, efficacy, and pharmacokinetic findings in advanced cancers. ASCO-SITC Clinical Immuno-Oncology Symposium. Volume 37. San Francisco: J Clin Oncol; 2019. p 12.

33. Papadopoulos KP, Lakhani N, Johnson ML, Park H, Wang D, Yap TA*, et al.* A study of REGN3767, an anti-LAG-3 antibody, alone and in combination with cemiplimab (REGN2810), an anti-PD1 antibody in advanced cancers. 2019 ASCO SITC Clinical Immuno-oncology Symposium. Volume 37. San Francisco: J Clin Oncol; 2019. p TPS41.

34. Tiragolumab Impresses in Multiple Trials. Cancer Discov **2020**;10(8):1086-7 doi 10.1158/2159-8290.CD-NB2020-063.

35. Oun R, Moussa YE, Wheate NJ. The side effects of platinum-based chemotherapy drugs: a review for chemists. Dalton Trans **2018**;47(19):6645-53 doi 10.1039/c8dt00838h.

36. Boyman O, Sprent J. The role of interleukin-2 during homeostasis and activation of the immune system. Nat Rev Immunol **2012**;12(3):180-90 doi 10.1038/nri3156.

37. Rosen LS, LoRusso P, Ma WW, Goldman JW, Weise A, Colevas AD*, et al.* A first-in-human phase I study to evaluate the MEK1/2 inhibitor, cobimetinib, administered daily in patients with advanced solid tumors. Invest New Drugs **2016**;34(5):604-13 doi 10.1007/s10637-016-0374-3.

38. Engel-Riedel W, Lowe J, Mattson P, Richard Trout J, Huhn RD, Gargano M*, et al.* A randomized, controlled trial evaluating the efficacy and safety of BTH1677 in combination with bevacizumab, carboplatin, and paclitaxel in first-line treatment of advanced non-small cell lung cancer. J Immunother Cancer **2018**;6(1):16 doi 10.1186/s40425-018-0324-z.

39. Naing A, Papadopoulos KP, Autio KA, Ott PA, Patel MR, Wong DJ*, et al.* Safety, Antitumor Activity, and Immune Activation of Pegylated Recombinant Human Interleukin-10 (AM0010) in Patients With Advanced Solid Tumors. J Clin Oncol **2016**;34(29):3562-9 doi 10.1200/JCO.2016.68.1106.

40. Bajor DL, Mick R, Riese MJ, Huang AC, Sullivan B, Richman LP*, et al.* Long-term outcomes of a phase I study of agonist CD40 antibody and CTLA-4 blockade in patients with metastatic melanoma. Oncoimmunology **2018**;7(10):e1468956 doi 10.1080/2162402X.2018.1468956.

41. Piechutta M, Berghoff AS. New emerging targets in cancer immunotherapy: the role of Cluster of Differentiation 40 (CD40/TNFR5). ESMO Open **2019**;4(Suppl 3):e000510 doi 10.1136/esmoopen-2019-000510.

42. Pol JG, Levesque S, Workenhe ST, Gujar S, Le Boeuf F, Clements DR*, et al.* Trial Watch: Oncolytic viro-immunotherapy of hematologic and solid tumors. Oncoimmunology **2018**;7(12):e1503032 doi 10.1080/2162402X.2018.1503032.

43. Sikic BI, Lakhani N, Patnaik A, Shah SA, Chandana SR, Rasco D*, et al.* First-in-Human, First-in-Class Phase I Trial of the Anti-CD47 Antibody Hu5F9-G4 in Patients With Advanced Cancers. J Clin Oncol **2019**;37(12):946-53 doi 10.1200/JCO.18.02018.

44. Singh H, Brave M, Beaver JA, Cheng J, Tang S, Zahalka E*, et al.* U.S. Food and Drug Administration Approval: Cabozantinib for the Treatment of Advanced Renal Cell Carcinoma. Clin Cancer Res **2017**;23(2):330-5 doi 10.1158/1078-0432.CCR-16-1073.
